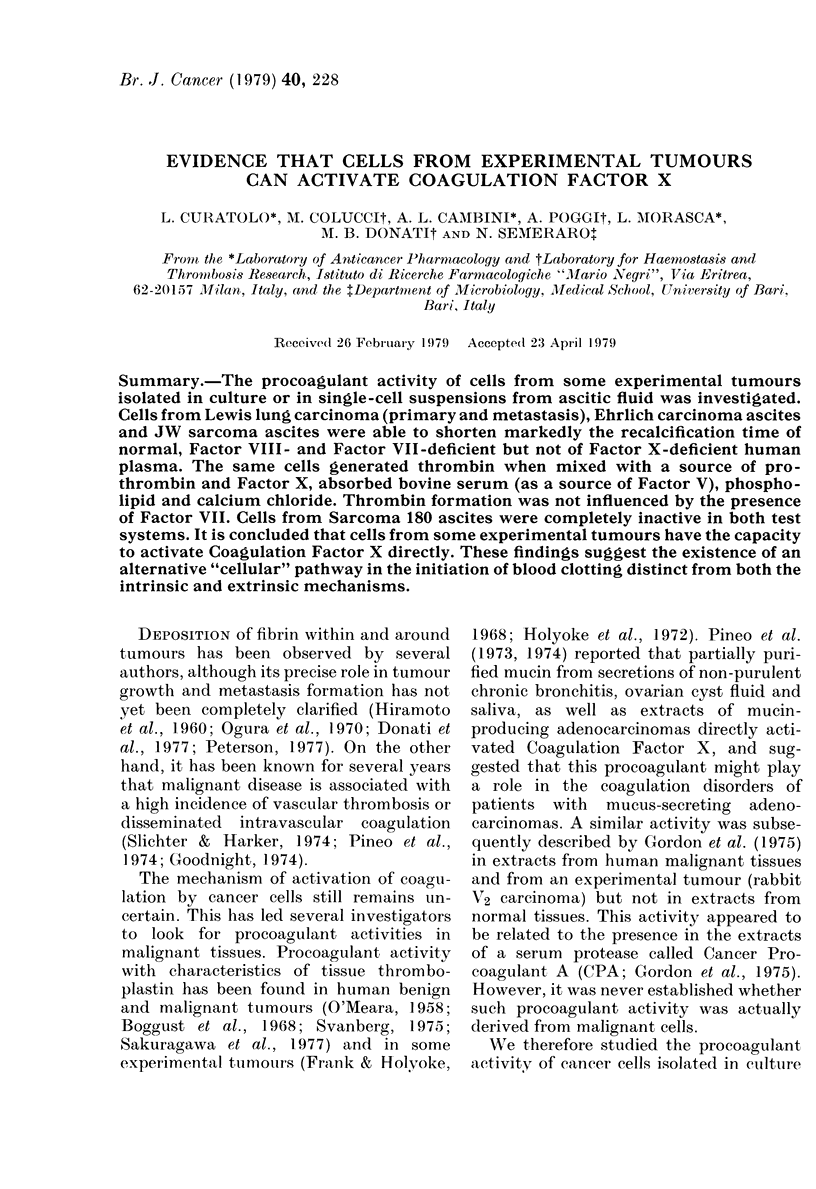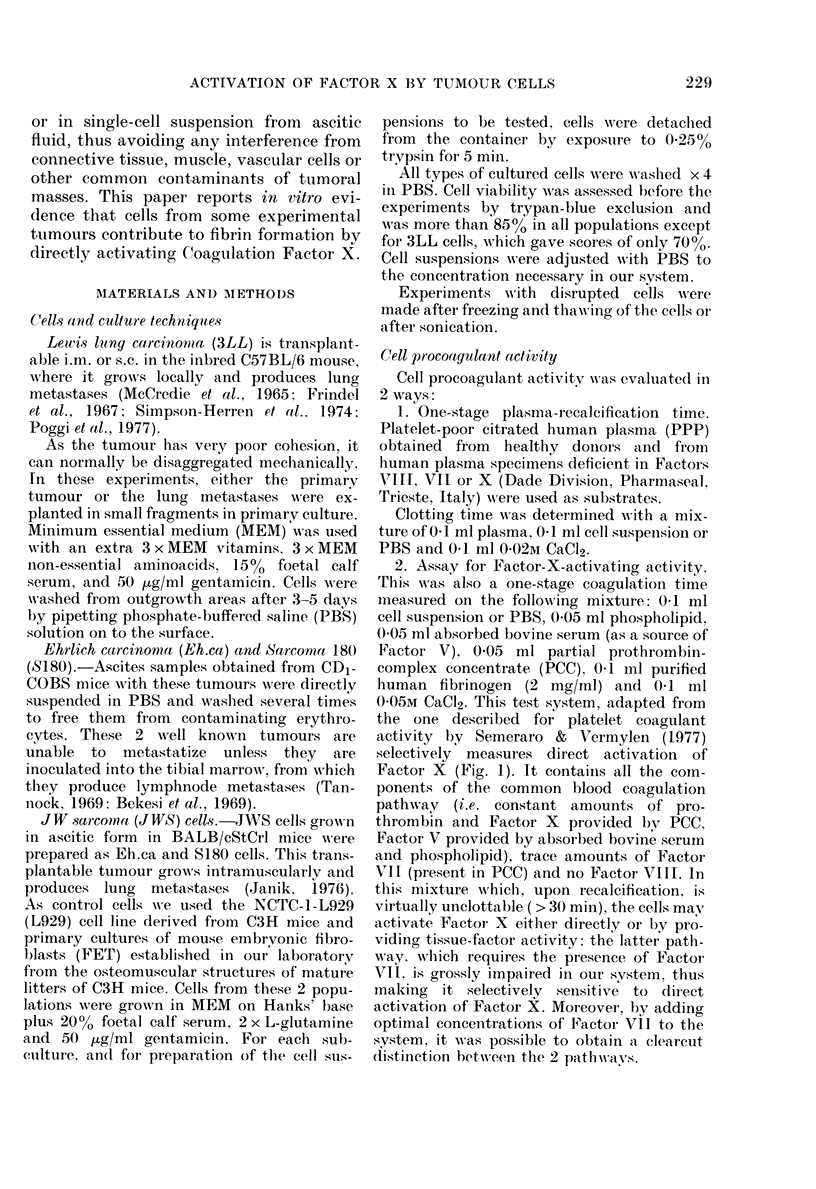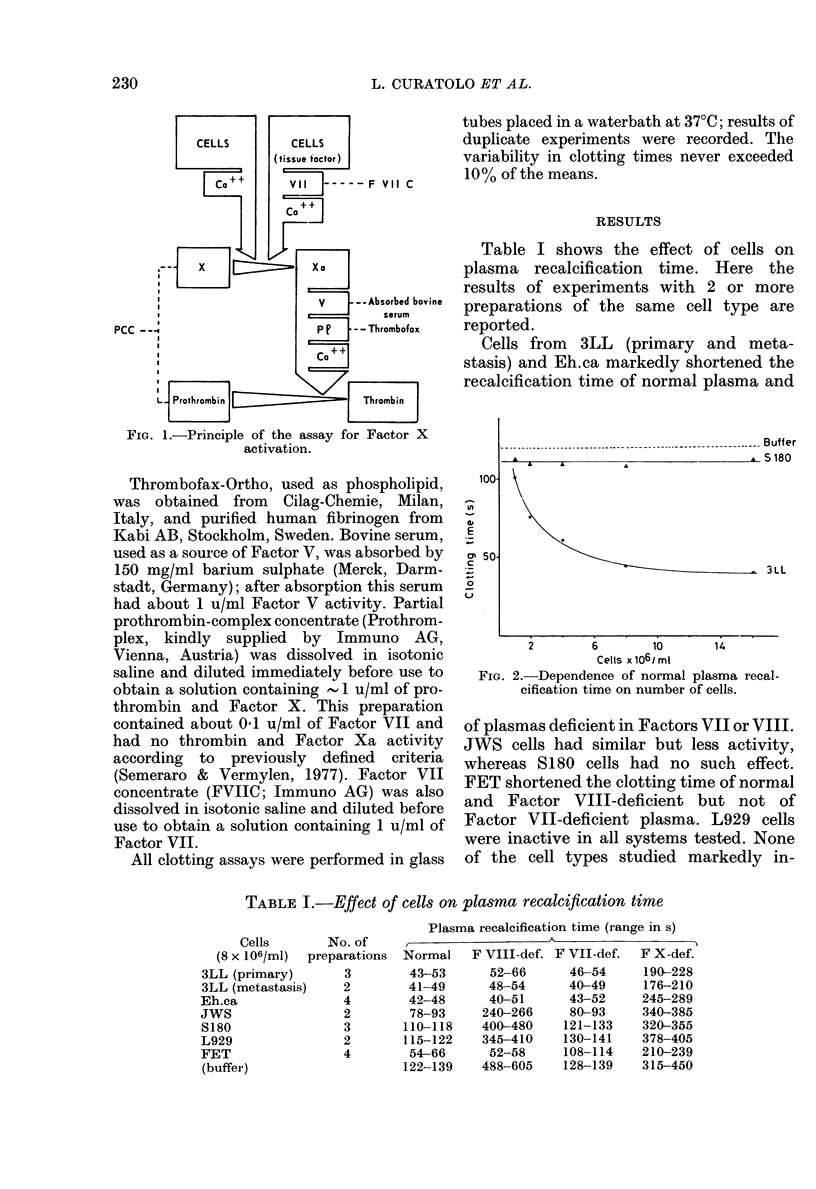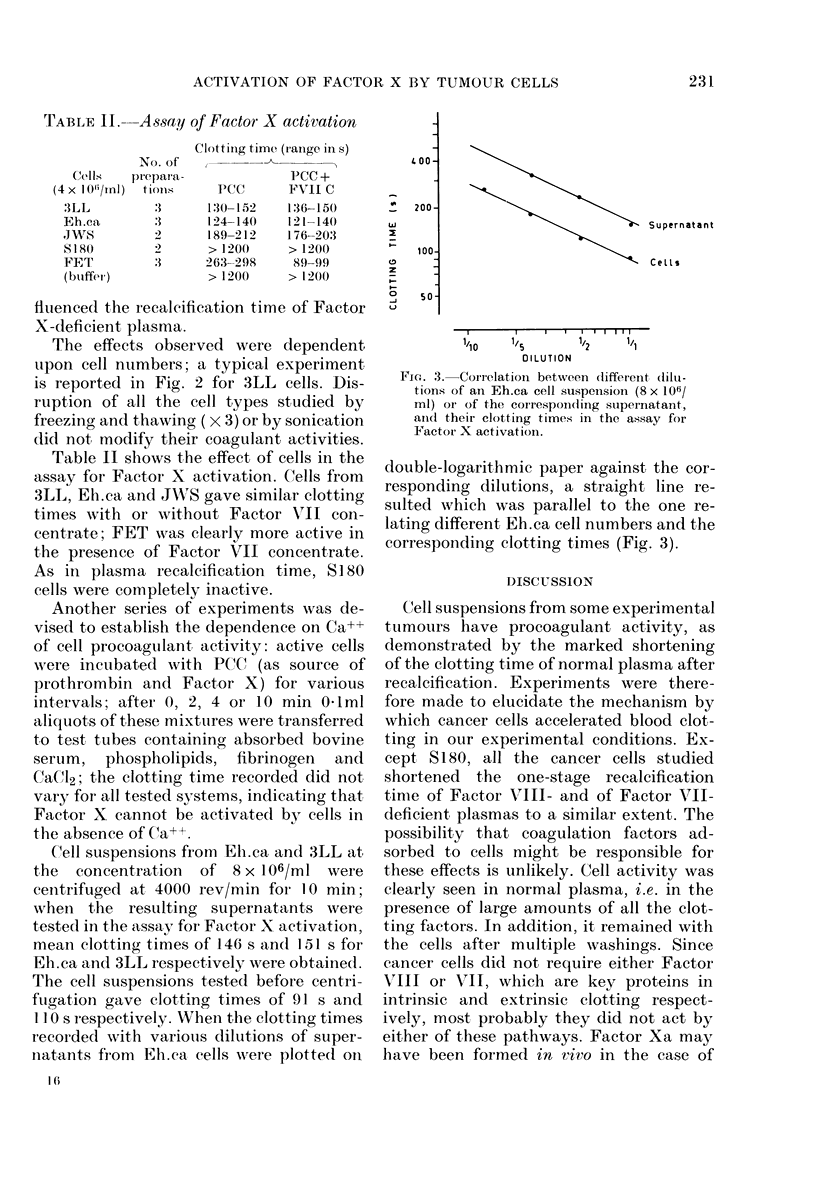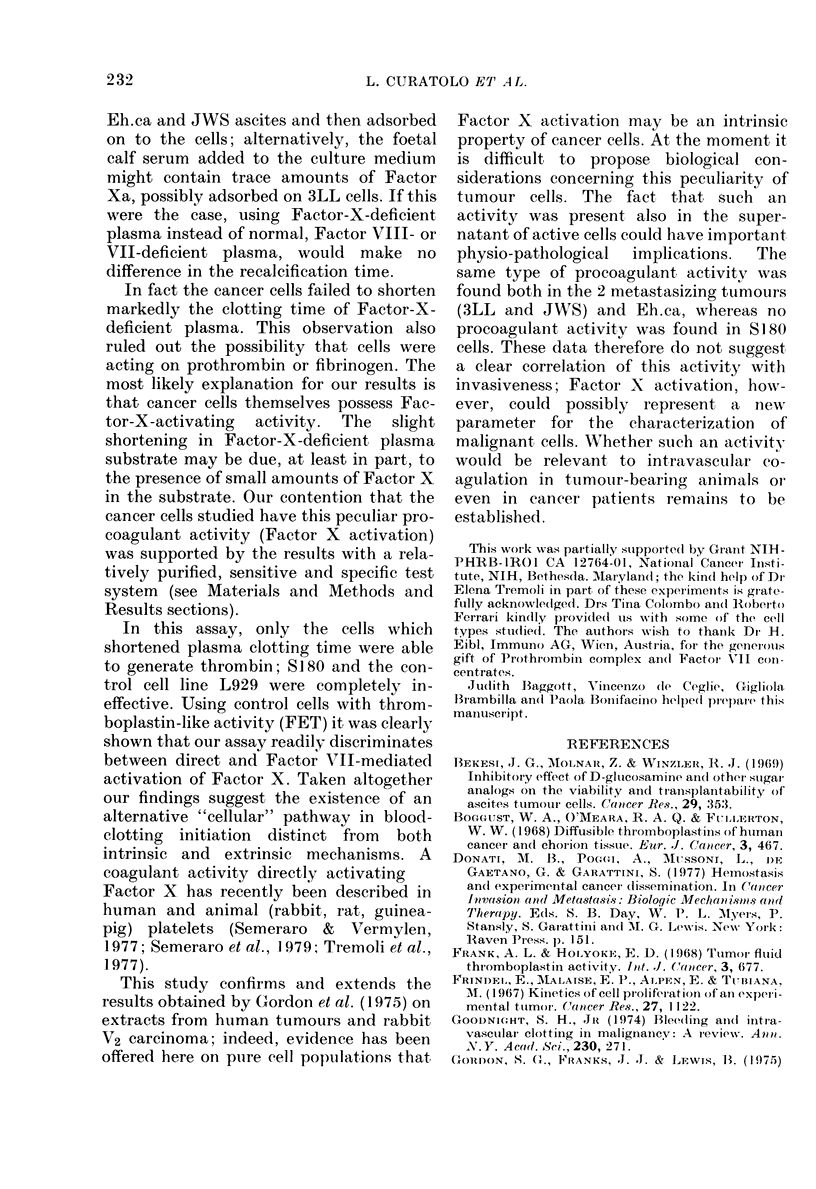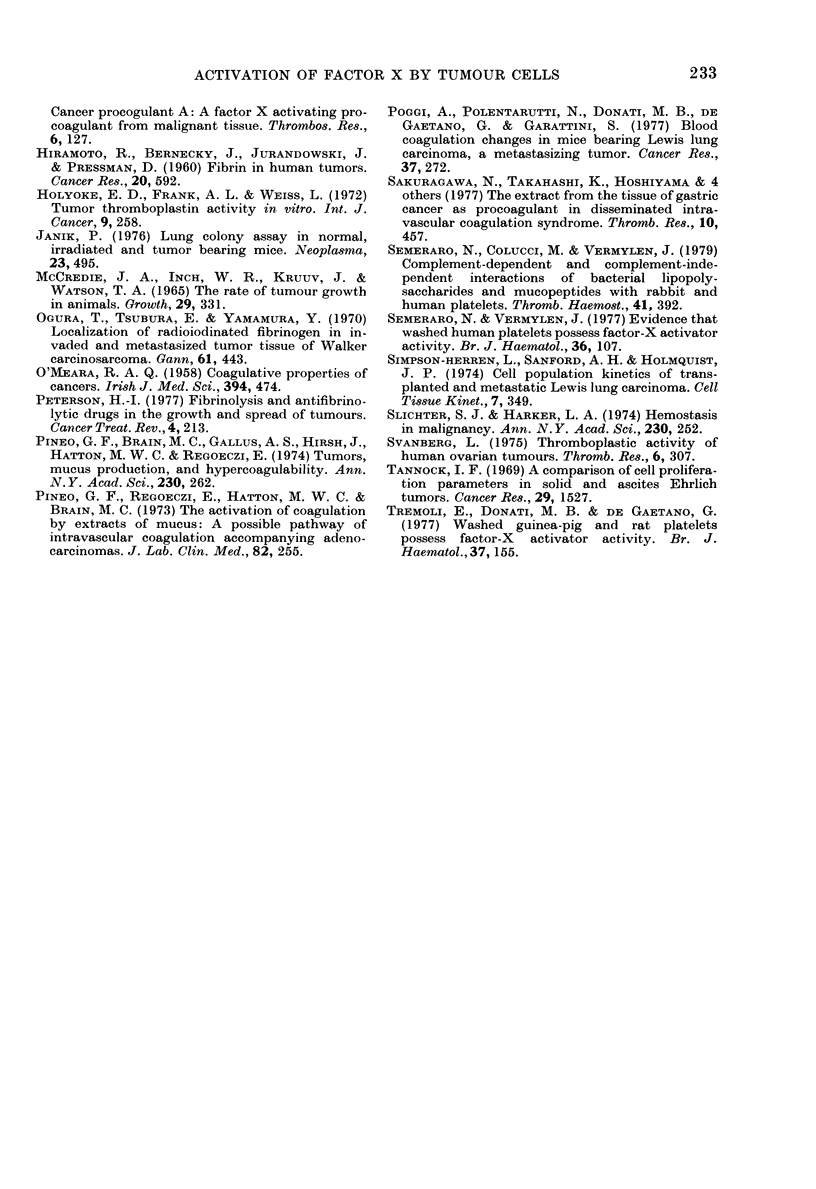# Evidence that cells from experimental tumours can activate coagulation factor X.

**DOI:** 10.1038/bjc.1979.170

**Published:** 1979-08

**Authors:** L. Curatolo, M. Colucci, A. L. Cambini, A. Poggi, L. Morasca, M. B. Donati, N. Semeraro

## Abstract

The procoagulant activity of cells from some experimental tumours isolated in culture or in single-cell suspensions from ascitic fluid was investigated. Cells from Lewis lung carcinoma (primary and metastasis), Ehrlich carcinoma ascites and JW sarcoma ascites were able to shorten markedly the recalcification time of normal, Factor VIII- and Factor VII-deficient but not of Factor X-deficient human plasma. The same cells generated thrombin when mixed with a source of prothrombin and Factor X, absorbed bovine serum (as a source of Factor V), phospholipid and calcium chloride. Thrombin formation was not influenced by the presence of Factor VII. Cells from Sarcoma 180 ascites were completely inactive in both test systems. It is concluded that cells from some experimental tumours have the capacity to activate Coagulation Factor X directly. These findings suggest the existence of an alternative "cellular" pathway in the initiation of blood clotting distinct from both the intrinsic and extrinsic mechanisms.


*Br. J. Cancer* (1979) **40**, 228

### EVIDENCE THAT CELLS FROM EXPERIMENTAL TUMOURS CAN ACTIVATE COAGULATION FACTOR X

L. CURATOLO*, M. COLUCCI†, A. L. CAMBINI*, A. POGGI†, L. MORASCA*, M. B. DONATI† AND N. SEMERARO‡

*From the *Laboratory of Anticancer Pharmacology and †Laboratory for Haemostasis and Thrombosis Research, Istituto di Ricerche Farmacologiche "Mario Negri", Via Eritrea,* 62-20157 *Milan, Italy, and the ‡Department of Microbiology, Medical School, University of Bari, Bari, Italy*

Received 26 February 1979 Accepted 23 April 1979

**Summary.—The procoagulant activity of cells from some experimental tumours isolated in culture or in single-cell suspensions from ascitic fluid was investigated. Cells from Lewis lung carcinoma (primary and metastasis), Ehrlich carcinoma ascites and JW sarcoma ascites were able to shorten markedly the recalcification time of normal, Factor VIII- and Factor VII-deficient but not of Factor X-deficient human plasma. The same cells generated thrombin when mixed with a source of prothrombin and Factor X, absorbed bovine serum (as a source of Factor V), phospholipid and calcium chloride. Thrombin formation was not influenced by the presence of Factor VII. Cells from Sarcoma 180 ascites were completely inactive in both test systems. It is concluded that cells from some experimental tumours have the capacity to activate Coagulation Factor X directly. These findings suggest the existence of an alternative "cellular" pathway in the initiation of blood clotting distinct from both the intrinsic and extrinsic mechanisms.**

DEPOSITION of fibrin within and around tumours has been observed by several authors, although its precise role in tumour growth and metastasis formation has not yet been completely clarified (Hiramoto *et al.*, 1960; Ogura *et al.*, 1970; Donati *et al.*, 1977; Peterson, 1977). On the other hand, it has been known for several years that malignant disease is associated with a high incidence of vascular thrombosis or disseminated intravascular coagulation (Slichter & Harker, 1974; Pineo *et al.*, 1974; Goodnight, 1974).

The mechanism of activation of coagulation by cancer cells still remains uncertain. This has led several investigators to look for procoagulant activities in malignant tissues. Procoagulant activity with characteristics of tissue thromboplastin has been found in human benign and malignant tumours (O'Meara, 1958; Boggust *et al.*, 1968; Svanberg, 1975; Sakuragawa *et al.*, 1977) and in some experimental tumours (Frank & Holyoke, 1968; Holyoke *et al.*, 1972). Pineo *et al.* (1973, 1974) reported that partially purified mucin from secretions of non-purulent chronic bronchitis, ovarian cyst fluid and saliva, as well as extracts of mucin-producing adenocarcinomas directly activated Coagulation Factor X, and suggested that this procoagulant might play a role in the coagulation disorders of patients with mucus-secreting adenocarcinomas. A similar activity was subsequently described by Gordon *et al.* (1975) in extracts from human malignant tissues and from an experimental tumour (rabbit $V_2$ carcinoma) but not in extracts from normal tissues. This activity appeared to be related to the presence in the extracts of a serum protease called Cancer Procoagulant A (CPA; Gordon *et al.*, 1975). However, it was never established whether such procoagulant activity was actually derived from malignant cells.

We therefore studied the procoagulant activity of cancer cells isolated in culture or in single-cell suspension from ascitic fluid, thus avoiding any interference from connective tissue, muscle, vascular cells or other common contaminants of tumoral masses. This paper reports *in vitro* evidence that cells from some experimental tumours contribute to fibrin formation by directly activating Coagulation Factor X.

### MATERIALS AND METHODS

### *Cells and culture techniques*

*Lewis lung carcinoma* (*3LL*) is transplantable i.m. or s.c. in the inbred C57BL/6 mouse, where it grows locally and produces lung metastases (McCredie *et al.*, 1965; Frindel *et al.*, 1967; Simpson-Herren *et al.*, 1974; Poggi *et al.*, 1977).

As the tumour has very poor cohesion, it can normally be disaggregated mechanically. In these experiments, either the primary tumour or the lung metastases were explanted in small fragments in primary culture. Minimum essential medium (MEM) was used with an extra 3 × MEM vitamins, 3 × MEM non-essential aminoacids, 15% foetal calf serum, and 50 μg/ml gentamicin. Cells were washed from outgrowth areas after 3–5 days by pipetting phosphate-buffered saline (PBS) solution on to the surface.

*Ehrlich carcinoma* (*Eh.ca*) *and Sarcoma* 180 (*S*180).—Ascites samples obtained from $CD_1$-COBS mice with these tumours were directly suspended in PBS and washed several times to free them from contaminating erythrocytes. These 2 well known tumours are unable to metastatize unless they are inoculated into the tibial marrow, from which they produce lymphnode metastases (Tannock, 1969; Bekesi *et al.*, 1969).

*JW sarcoma* (*JWS*) *cells*.—JWS cells grown in ascitic form in BALB/cStCrl mice were prepared as Eh.ca and S180 cells. This transplantable tumour grows intramuscularly and produces lung metastases (Janik, 1976). As control cells we used the NCTC-1-L929 (L929) cell line derived from C3H mice and primary cultures of mouse embryonic fibroblasts (FET) established in our laboratory from the osteomuscular structures of mature litters of C3H mice. Cells from these 2 populations were grown in MEM on Hanks' base plus 20% foetal calf serum, 2 × L-glutamine and 50 μg/ml gentamicin. For each subculture, and for preparation of the cell suspensions to be tested, cells were detached from the container by exposure to 0·25% trypsin for 5 min.

All types of cultured cells were washed × 4 in PBS. Cell viability was assessed before the experiments by trypan-blue exclusion and was more than 85% in all populations except for 3LL cells, which gave scores of only 70%. Cell suspensions were adjusted with PBS to the concentration necessary in our system.

Experiments with disrupted cells were made after freezing and thawing of the cells or after sonication.

### *Cell procoagulant activity*

Cell procoagulant activity was evaluated in 2 ways:

1. One-stage plasma-recalcification time. Platelet-poor citrated human plasma (PPP) obtained from healthy donors and from human plasma specimens deficient in Factors VIII, VII or X (Dade Division, Pharmaseal, Trieste, Italy) were used as substrates.

Clotting time was determined with a mixture of 0·1 ml plasma, 0·1 ml cell suspension or PBS and 0·1 ml 0·02M $CaCl_2$.

2. Assay for Factor-X-activating activity. This was also a one-stage coagulation time measured on the following mixture: 0·1 ml cell suspension or PBS, 0·05 ml phospholipid, 0·05 ml absorbed bovine serum (as a source of Factor V), 0·05 ml partial prothrombin-complex concentrate (PCC), 0·1 ml purified human fibrinogen (2 mg/ml) and 0·1 ml 0·05M $CaCl_2$. This test system, adapted from the one described for platelet coagulant activity by Semeraro & Vermylen (1977) selectively measures direct activation of Factor X (Fig. 1). It contains all the components of the common blood coagulation pathway (*i.e.* constant amounts of prothrombin and Factor X provided by PCC, Factor V provided by absorbed bovine serum and phospholipid), trace amounts of Factor VII (present in PCC) and no Factor VIII. In this mixture which, upon recalcification, is virtually unclottable ( > 30 min), the cells may activate Factor X either directly or by providing tissue-factor activity: the latter pathway, which requires the presence of Factor VII, is grossly impaired in our system, thus making it selectively sensitive to direct activation of Factor X. Moreover, by adding optimal concentrations of Factor VII to the system, it was possible to obtain a clearcut distinction between the 2 pathways.

Fig. 1.—Principle of the assay for Factor X activation.

Thrombofax-Ortho, used as phospholipid, was obtained from Cilag-Chemie, Milan, Italy, and purified human fibrinogen from Kabi AB, Stockholm, Sweden. Bovine serum, used as a source of Factor V, was absorbed by 150 mg/ml barium sulphate (Merck, Darmstadt, Germany); after absorption this serum had about 1 u/ml Factor V activity. Partial prothrombin-complex concentrate (Prothromplex, kindly supplied by Immuno AG, Vienna, Austria) was dissolved in isotonic saline and diluted immediately before use to obtain a solution containing ~1 u/ml of prothrombin and Factor X. This preparation contained about 0·1 u/ml of Factor VII and had no thrombin and Factor Xa activity according to previously defined criteria (Semeraro & Vermylen, 1977). Factor VII concentrate (FVIIC; Immuno AG) was also dissolved in isotonic saline and diluted before use to obtain a solution containing 1 u/ml of Factor VII.

All clotting assays were performed in glass tubes placed in a waterbath at 37°C; results of duplicate experiments were recorded. The variability in clotting times never exceeded 10% of the means.

### RESULTS

Table I shows the effect of cells on plasma recalcification time. Here the results of experiments with 2 or more preparations of the same cell type are reported.

Cells from 3LL (primary and metastasis) and Eh.ca markedly shortened the recalcification time of normal plasma and of plasmas deficient in Factors VII or VIII. JWS cells had similar but less activity, whereas S180 cells had no such effect. FET shortened the clotting time of normal and Factor VIII-deficient but not of Factor VII-deficient plasma. L929 cells were inactive in all systems tested. None of the cell types studied markedly in-

Fig. 2.—Dependence of normal plasma recalcification time on number of cells.

Table I.—*Effect of cells on plasma recalcification time*

| Cells ($8 \times 10^6$/ml) | No. of preparations | Plasma recalcification time (range in s): Normal | F VIII-def. | F VII-def. | F X-def. |
|---|---|---|---|---|---|
| 3LL (primary) | 3 | 43–53 | 52–66 | 46–54 | 190–228 |
| 3LL (metastasis) | 2 | 41–49 | 48–54 | 40–49 | 176–210 |
| Eh.ca | 4 | 42–48 | 40–51 | 43–52 | 245–289 |
| JWS | 2 | 78–93 | 240–266 | 80–93 | 340–385 |
| S180 | 3 | 110–118 | 400–480 | 121–133 | 320–355 |
| L929 | 2 | 115–122 | 345–410 | 130–141 | 378–405 |
| FET | 4 | 54–66 | 52–58 | 108–114 | 210–239 |
| (buffer) | | 122–139 | 488–605 | 128–139 | 315–450 |

TABLE II.—*Assay of Factor X activation*

| Cells ($4 \times 10^6$/ml) | No. of preparations | Clotting time (range in s) PCC | PCC + FVII C |
|---|---|---|---|
| 3LL | 3 | 130–152 | 136–150 |
| Eh.ca | 3 | 124–140 | 121–140 |
| JWS | 2 | 189–212 | 176–203 |
| S180 | 2 | > 1200 | > 1200 |
| FET | 3 | 263–298 | 89–99 |
| (buffer) | | > 1200 | > 1200 |

fluenced the recalcification time of Factor X-deficient plasma.

The effects observed were dependent upon cell numbers; a typical experiment is reported in Fig. 2 for 3LL cells. Disruption of all the cell types studied by freezing and thawing ($\times 3$) or by sonication did not modify their coagulant activities.

Table II shows the effect of cells in the assay for Factor X activation. Cells from 3LL, Eh.ca and JWS gave similar clotting times with or without Factor VII concentrate; FET was clearly more active in the presence of Factor VII concentrate. As in plasma recalcification time, S180 cells were completely inactive.

Another series of experiments was devised to establish the dependence on $Ca^{++}$ of cell procoagulant activity: active cells were incubated with PCC (as source of prothrombin and Factor X) for various intervals; after 0, 2, 4 or 10 min 0·1ml aliquots of these mixtures were transferred to test tubes containing absorbed bovine serum, phospholipids, fibrinogen and $CaCl_2$; the clotting time recorded did not vary for all tested systems, indicating that Factor X cannot be activated by cells in the absence of $Ca^{++}$.

Cell suspensions from Eh.ca and 3LL at the concentration of $8 \times 10^6$/ml were centrifuged at 4000 rev/min for 10 min; when the resulting supernatants were tested in the assay for Factor X activation, mean clotting times of 146 s and 151 s for Eh.ca and 3LL respectively were obtained. The cell suspensions tested before centrifugation gave clotting times of 91 s and 110 s respectively. When the clotting times recorded with various dilutions of supernatants from Eh.ca cells were plotted on

FIG. 3.—Correlation between different dilutions of an Eh.ca cell suspension ($8 \times 10^6$/ ml) or of the corresponding supernatant, and their clotting times in the assay for Factor X activation.

double-logarithmic paper against the corresponding dilutions, a straight line resulted which was parallel to the one relating different Eh.ca cell numbers and the corresponding clotting times (Fig. 3).

### DISCUSSION

Cell suspensions from some experimental tumours have procoagulant activity, as demonstrated by the marked shortening of the clotting time of normal plasma after recalcification. Experiments were therefore made to elucidate the mechanism by which cancer cells accelerated blood clotting in our experimental conditions. Except S180, all the cancer cells studied shortened the one-stage recalcification time of Factor VIII- and of Factor VII-deficient plasmas to a similar extent. The possibility that coagulation factors adsorbed to cells might be responsible for these effects is unlikely. Cell activity was clearly seen in normal plasma, *i.e.* in the presence of large amounts of all the clotting factors. In addition, it remained with the cells after multiple washings. Since cancer cells did not require either Factor VIII or VII, which are key proteins in intrinsic and extrinsic clotting respectively, most probably they did not act by either of these pathways. Factor Xa may have been formed *in vivo* in the case of

Eh.ca and JWS ascites and then adsorbed on to the cells; alternatively, the foetal calf serum added to the culture medium might contain trace amounts of Factor Xa, possibly adsorbed on 3LL cells. If this were the case, using Factor-X-deficient plasma instead of normal, Factor VIII- or VII-deficient plasma, would make no difference in the recalcification time.

In fact the cancer cells failed to shorten markedly the clotting time of Factor-X-deficient plasma. This observation also ruled out the possibility that cells were acting on prothrombin or fibrinogen. The most likely explanation for our results is that cancer cells themselves possess Factor-X-activating activity. The slight shortening in Factor-X-deficient plasma substrate may be due, at least in part, to the presence of small amounts of Factor X in the substrate. Our contention that the cancer cells studied have this peculiar procoagulant activity (Factor X activation) was supported by the results with a relatively purified, sensitive and specific test system (see Materials and Methods and Results sections).

In this assay, only the cells which shortened plasma clotting time were able to generate thrombin; S180 and the control cell line L929 were completely ineffective. Using control cells with thromboplastin-like activity (FET) it was clearly shown that our assay readily discriminates between direct and Factor VII-mediated activation of Factor X. Taken altogether our findings suggest the existence of an alternative "cellular" pathway in blood-clotting initiation distinct from both intrinsic and extrinsic mechanisms. A coagulant activity directly activating Factor X has recently been described in human and animal (rabbit, rat, guinea-pig) platelets (Semeraro & Vermylen, 1977; Semeraro *et al.*, 1979; Tremoli *et al.*, 1977).

This study confirms and extends the results obtained by Gordon *et al.* (1975) on extracts from human tumours and rabbit $V_2$ carcinoma; indeed, evidence has been offered here on pure cell populations that Factor X activation may be an intrinsic property of cancer cells. At the moment it is difficult to propose biological considerations concerning this peculiarity of tumour cells. The fact that such an activity was present also in the supernatant of active cells could have important physio-pathological implications. The same type of procoagulant activity was found both in the 2 metastasizing tumours (3LL and JWS) and Eh.ca, whereas no procoagulant activity was found in S180 cells. These data therefore do not suggest a clear correlation of this activity with invasiveness; Factor X activation, however, could possibly represent a new parameter for the characterization of malignant cells. Whether such an activity would be relevant to intravascular coagulation in tumour-bearing animals or even in cancer patients remains to be established.

This work was partially supported by Grant NIH-PHRB-1R01 CA 12764-01, National Cancer Institute, NIH, Bethesda. Maryland; the kind help of Dr Elena Tremoli in part of these experiments is gratefully acknowledged. Drs Tina Colombo and Roberto Ferrari kindly provided us with some of the cell types studied. The authors wish to thank Dr H. Eibl, Immuno AG, Wien, Austria, for the generous gift of Prothrombin complex and Factor VII concentrates.

Judith Baggott, Vincenzo de Ceglie, Gigliola Brambilla and Paola Bonifacino helped prepare this manuscript.

### REFERENCES

BEKESI, J. G., MOLNAR, Z. & WINZLER, R. J. (1969) Inhibitory effect of D-glucosamine and other sugar analogs on the viability and transplantability of ascites tumour cells. *Cancer Res.*, **29**, 353.

BOGGUST, W. A., O'MEARA, R. A. Q. & FULLERTON, W. W. (1968) Diffusible thromboplastins of human cancer and chorion tissue. *Eur. J. Cancer*, **3**, 467.

DONATI, M. B., POGGI, A., MUSSONI, L., DE GAETANO, G. & GARATTINI, S. (1977) Hemostasis and experimental cancer dissemination. In *Cancer Invasion and Metastasis: Biologic Mechanisms and Therapy*. Eds. S. B. Day, W. P. L. Myers, P. Stansly, S. Garattini and M. G. Lewis. New York: Raven Press. p. 151.

FRANK, A. L. & HOLYOKE, E. D. (1968) Tumor fluid thromboplastin activity. *Int. J. Cancer*, **3**, 677.

FRINDEL, E., MALAISE, E. P., ALPEN, E. & TUBIANA, M. (1967) Kinetics of cell proliferation of an experimental tumor. *Cancer Res.*, **27**, 1122.

GOODNIGHT, S. H., JR (1974) Bleeding and intravascular clotting in malignancy: A review. *Ann. N.Y. Acad. Sci.*, **230**, 271.

GORDON, S. G., FRANKS, J. J. & LEWIS, B. (1975)

Cancer procogulant A: A factor X activating procoagulant from malignant tissue. *Thrombos. Res.*, **6**, 127.

HIRAMOTO, R., BERNECKY, J., JURANDOWSKI, J. & PRESSMAN, D. (1960) Fibrin in human tumors. *Cancer Res.*, **20**, 592.

HOLYOKE, E. D., FRANK, A. L. & WEISS, L. (1972) Tumor thromboplastin activity *in vitro*. *Int. J. Cancer*, **9**, 258.

JANIK, P. (1976) Lung colony assay in normal, irradiated and tumor bearing mice. *Neoplasma*, **23**, 495.

MCCREDIE, J. A., INCH, W. R., KRUUV, J. & WATSON, T. A. (1965) The rate of tumour growth in animals. *Growth*, **29**, 331.

OGURA, T., TSUBURA, E. & YAMAMURA, Y. (1970) Localization of radioiodinated fibrinogen in invaded and metastasized tumor tissue of Walker carcinosarcoma. *Gann*, **61**, 443.

O'MEARA, R. A. Q. (1958) Coagulative properties of cancers. *Irish J. Med. Sci.*, **394**, 474.

PETERSON, H.-I. (1977) Fibrinolysis and antifibrinolytic drugs in the growth and spread of tumours. *Cancer Treat. Rev.*, **4**, 213.

PINEO, G. F., BRAIN, M. C., GALLUS, A. S., HIRSH, J., HATTON, M. W. C. & REGOECZI, E. (1974) Tumors, mucus production, and hypercoagulability. *Ann. N.Y. Acad. Sci.*, **230**, 262.

PINEO, G. F., REGOECZI, E., HATTON, M. W. C. & BRAIN, M. C. (1973) The activation of coagulation by extracts of mucus: A possible pathway of intravascular coagulation accompanying adenocarcinomas. *J. Lab. Clin. Med.*, **82**, 255.

POGGI, A., POLENTARUTTI, N., DONATI, M. B., DE GAETANO, G. & GARATTINI, S. (1977) Blood coagulation changes in mice bearing Lewis lung carcinoma, a metastasizing tumor. *Cancer Res.*, **37**, 272.

SAKURAGAWA, N., TAKAHASHI, K., HOSHIYAMA & 4 others (1977) The extract from the tissue of gastric cancer as procoagulant in disseminated intravascular coagulation syndrome. *Thromb. Res.*, **10**, 457.

SEMERARO, N., COLUCCI, M. & VERMYLEN, J. (1979) Complement-dependent and complement-independent interactions of bacterial lipopolysaccharides and mucopeptides with rabbit and human platelets. *Thromb. Haemost.*, **41**, 392.

SEMERARO, N. & VERMYLEN, J. (1977) Evidence that washed human platelets possess factor-X activator activity. *Br. J. Haematol.*, **36**, 107.

SIMPSON-HERREN, L., SANFORD, A. H. & HOLMQUIST, J. P. (1974) Cell population kinetics of transplanted and metastatic Lewis lung carcinoma. *Cell Tissue Kinet.*, **7**, 349.

SLICHTER, S. J. & HARKER, L. A. (1974) Hemostasis in malignancy. *Ann. N.Y. Acad. Sci.*, **230**, 252.

SVANBERG, L. (1975) Thromboplastic activity of human ovarian tumours. *Thromb. Res.*, **6**, 307.

TANNOCK, I. F. (1969) A comparison of cell proliferation parameters in solid and ascites Ehrlich tumors. *Cancer Res.*, **29**, 1527.

TREMOLI, E., DONATI, M. B. & DE GAETANO, G. (1977) Washed guinea-pig and rat platelets possess factor-X activator activity. *Br. J. Haematol.*, **37**, 155.